# Global burden and inequality of iron deficiency: findings from the Global Burden of Disease datasets 1990–2017

**DOI:** 10.1186/s12937-022-00771-3

**Published:** 2022-03-18

**Authors:** Mengying Wang, He Gao, Jianing Wang, Chenliang Cao, Xiaoling Ying, Yingming Wei, Zhiying Yu, Jie Shao, Hengjin Dong, Min Yang

**Affiliations:** 1 Department of Nutrition and Food Hygiene, School of Public Health, Zhejiang University School of Medicine, Hangzhou, China; 2 Department of Big Data in Health Science School of Public Health, and Center of Clinical Big Data and Analytics of The Second Affiliated Hospital, Zhejiang University School of Medicine, Hangzhou, China; 3 Department of Nutrition, The Second Affiliated Hospital of Zhejiang University School of Medicine, Linping Campus, Hangzhou, China; 4 Nutritional Department, Chongqing Health Center for Women and Children, Chongqing, China; 5 Sports Nutrition Center, National Institute of Sports Medicine, Beijing, China; 6 Department of Periodontology, The Second Affiliated Hospital of Zhejiang University School of Medicine, Hangzhou, China; 7 Department of Pediatric Health Care Children’s Hospital, Zhejiang University School of Medicine National Clinical Research Center for Child Health, Hangzhou, China; 8 Center for Health Policy Studies, School of Public Health, School of Medicine, Zhejiang University, Hangzhou, China

**Keywords:** Sex difference, Micronutrient deficiency, Iron deficiency, Global burden of disease, Equality, Socioeconomic status

## Abstract

**Background:**

Iron deficiency (ID) impairs patient physical activity, recognition and life quality, which is difficult to perceive but should not be underestimated. Worldwide efforts have been made to lower ID burden, however, whether it decreased equally in different regions and sexes is unclear. This study is to examine regional and sex inequalities in global ID from 1990 to 2017.

**Methods:**

We conducted a longitudinal, comparative burden-of-disease study. Disability-adjusted life-years (DALYs) of ID were obtained from Global Burden of Disease Report 2017. Human Development Index (HDI) data were obtained from Human Development Report 2017. Gini coefficient and the concentration index were calculated to assess the equities in global burden of ID.

**Results:**

A downward trend of global ID burden (from 569.3 (95% Uncertainty Interval [UI]: 387.8–815.6) to 403.0 (95% UI: 272.4–586.6), *p* < 0.001), age-adjusted DALYs per 100,000 population) but an uptrend of its inequalities (from 0.366 to 0.431, *p* < 0.001, Gini coefficients) was observed between 1990 and 2017. ID burden was heavier in women than that in men ([age-adjusted DALYs per 100,000 population from 742.2 to 514.3] vs [from 398.5 to 291.9]), but its inequalities were higher in men since 1990. The between-sex gap of ID burden was narrowed with higher HDI (*β* =  − 364.11, *p* < 0.001). East Asia & Pacific and South Asia regions made a big stride for ID control in both sexes over decades [age-adjusted DALYs per 100,000 population from 378.7 (95% UI: 255.8–551.7) in 1990 to 138.9 (95%UI: 91.8–206.5) in 2017], while a heavy burden among Sub-Saharan African men was persistent[age-adjusted DALYs per 100,000 population, 572.5 (95% UI: 385.3–815) in 1990 and 562.6 (95% UI: 367.9–833.3) in 2017].

**Conclusions:**

Redistributing attention and resources to help countries with low HDI, especially take care of women with low socioeconomic status (SES) and men under high ID burden may help hold back the expanding ID inequality.

**Supplementary Information:**

The online version contains supplementary material available at 10.1186/s12937-022-00771-3.

## Introduction

Iron deficiency (ID) refers to the reduction of iron stores that precedes overt iron-deficiency anemia or persists without progression. [1]. Severe ID without treatment would progress to anemia [1]. Although the prevalence of ID anemia has recently declined to some extent, ID continues to be among the top causes of anemia worldwide [1]. ID impairs physical activity, cognitive performance, and quality of life [2], and increases societal cost [3, 4]. The latest Global Burden of Disease Study 2017 (GBD 2017) reported results from 195 countries and revealed that ID contributed to around 30 million Disability-adjusted life-years (DALYs) in 2017, which is the highest amongst other nutritional deficiencies [5].

However, the distribution of age-standardized DALYs of ID varied among countries of different income levels, suggesting an ID inequity problem at global level. Health inequity usually results from unequal distribution of power, prestige, and resources among groups in society [6]. Throughout the Sustainable Development Goals (SDGs), the aim of “health equity” is highlighted. Human development index (HDI) is a summary measure of average achievement in key dimensions of human development, and is often used to measure national socioeconomic status (SES) [7]. Emadi et al*.* found that communicable, maternal, neonatal, and nutritional diseases burden were more concentrated in countries with low-HDI [8]. To date, few studies examined the ID inequity problem among geographic regions with different HDI at a global level.

Moreover, even within the same geographic region, the distribution of ID between men and women can be different [9]. GBD 2017 exhibited and compared between-sex DALYs of ID in 1990, 2007 and 2017, pointed out that age-standardized DALY rates due to ID for adult women were almost twice as large as that for men [5]. Women of reproductive age are more likely to suffer from ID because of their increased iron requirements due to menstruation, pregnancy, and breastfeeding [10]. Findings from studies of better developed countries like India [11], Cambodia [12], and Mexico [13] indicated that domestic inequalities of ID among women. Yang et al*.* found persistent anemic inequalities among non-pregnant women in less developed African countries like Sierra Leone and Benin [14]. Despite the intense research on women, we found less attention has been put on men. Among the few studies that were conducted in men, the prevalence of anemia in boys and adolescent men was reported as 89.9% in India [15], 56.3% in Nepal [16], and about 88.5% in in Egypt [17]. There are far fewer studies in adult men than in women.

Therefore, in the current study, we aimed to first evaluate the ID burden globally, across geographic regions, and across countries with different HDI, and second, to examined ID burden between men and women. Our findings will provide a comprehensive description of the ID burden and provide evidence for improvement in health policies regarding ID inequity.

## Methods

### Study design and the aim

We conducted a comparative burden-of-disease study with the longitudinal GBD 1990–2017 data. Using data from GBD 1990–2017, we first evaluated the ID burden globally, across geographic regions, and across countries with different HDI. Second, we examined ID burden between men and women within the same geographic region or within countries with similar HDI. Last, we assessed the change in ID burden over decades in both men and women globally.

### Age-standardized DALYs

Disability adjusted life years (DALYs) is commonly used to identify disease burden; it quantifies the health loss of acute and chronic disease and injury which combines mortality, morbidity, and disability to measure the burden of diseases, injuries, and risk factors, and age-standardized DALYs was considered as a proper metric for comparison across countries [18]. We used rates of DALYs (DALYs per 100 000 population) for better comparability across countries of varying size.

Global burden of dietary ID was estimated in terms of age-standardized DALYs per 100 000 population in GBD 2017, which included 195 countries since 1990. These data are disaggregated by sex, age group, country (in some cases by state), and geographic region, and are open source and available for download from the GBD Results Tool. Detailed descriptions of the methods and approach used for the GBD estimation have been previously described [5]. Briefly, we downloaded dataset including: (1) global age-standardized DALYs rates from 1990 to 2017; (2) national age-standardized DALYs rates from 1990 to 2017; (3) sex-specific age-standardized DALYs rates from 1990 to 2017; and (4) sex-specific age-standardized DALYs rates changes compare 1990 with 2017 for World Bank regions; (5) Sex-specific DALYs of age groups in 2017.

These estimates are modelled based on a large number of input sources (eg, country-level census, disease registries, and local and international surveys) and is updated annually, detailed data input source can be found at GBD 2017 Data Input Sources Tool (http://ghdx.healthdata.org/gbd-2017/data-input-sources) [19], which contains relevant metadata about the input sources as suggested in the Guidelines for Accurate and Transparent Health Estimates Reporting (GATHER).

### Human Development Index

Calculation of the HDI combines four major indicators: life expectancy at birth, expected years of schooling, mean years of schooling, and gross national income per capita for a standard of living [7]. The HDI score ranges from 0 to 1: a higher value indicates a higher socioeconomic development level [7].

Sex and country disaggregated HDI data from 1990 to 2017 was downloaded from Human Development Report 2017 releases by the United Nations Development Programme (http://hdr.undp.org/en/content/download-data) [20]. The data source input to calculate HDI can be downloaded freely (http://data.un.org/) [21]. HDI data were available for 185 countries in 2017. We categorized countries into four categories based on their HDI as low (< 0.550), medium (0.550–0.699), high (0.700–0.799), and very high (≥ 0.80) [7].

### Measures of health inequity

The Gini coefficient and concentration index were used to measure health inequality. The Gini coefficient is a description of statistical dispersion intended to represent the disease burden distribution of regions and territories [22, 23]. A Gini coefficient of 0 represents perfect equality, while that of 1 represents the opposite situation. We computed Gini coefficients based on the age-standardized DALY rates. The concentration index is a measure of socioeconomic-related inequality extent, ranked by HDI: a negative value indicates that the ID-induced disease burden is higher among low-HDI countries.

### Statistical analysis

First, we described and reported the ID burden in different geographic regions and among men and women. Trend test was conducted by linear regression. The change in the rate of age-standardized DALYs was calculated as [(Age-standardized DALY rates in 2017- Age-standardized DALY rates in 1990)/ Age-standardized DALY rates in 1990]. To provide an overview of the ID burden in women and men from another perspective, we compared the ID burden in terms of DALYs per 100,000 by age groups.

Second, the Kruskal–Wallis test was performed to evaluate differences in age-standardized DALY rates among different HDI groups in 2017, and the Nemenyi test was used for post-hoc between-group comparisons. The Mann–Whitney U test was adopted to compare the differences in age-standardized DALY rates between men and women. The gap of age-standardized DALYs was calculated as the ID burden in women subtracting the ID burden in men. We then used linear regression to explore the relationship of the gap with HDI. We further grouped the countries by both HDI and geographic regions, and examined the associations between GDP growth and the decrease in the age-standardized DALY rates.

Third, we used time series imputation to impute missing HDI and used dynamic time warping to classify the HDI trajectories for 152 countries into three clusters as low, moderate, and high.

Last, we conducted sex-specific trend analyses on Gini coefficient, and concentration index from 1990 to 2017 by linear regression.

Analyses and plotting were conducted using R version 3.5.2. The Gini coefficient and Concentration Index was calculated with package “*IC2*”, time series imputation were conducted using package “*imputeTS*” [24], and dynamic time warping was conducted with the package “*dtwcluster*” [25]. All tests were two-tailed, and a *p* < 0.05 was considered as statistically significant.

## Results

### ID burden globally and in geographic regions

**Fig. 1:**
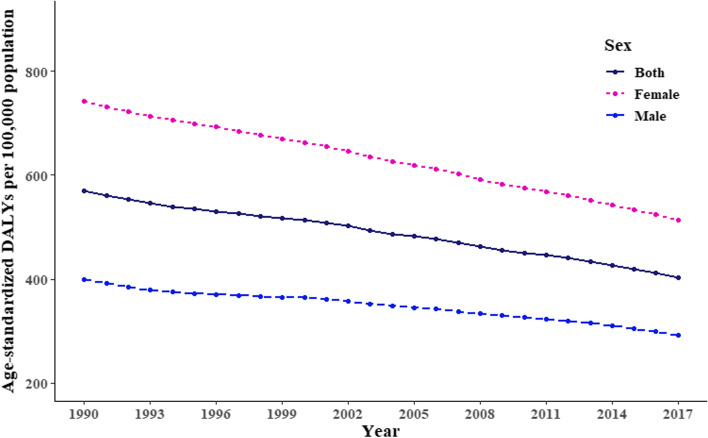
Trends in global burden of ID from 1990 to 2017, in terms of age-standardized DALY rates in both sexes

Between 1990 and 2017, we observed a downward trend (*p* < 0.001) of global age-standardized DALY rates, falling from 569.3 (95% uncertainty interval [UI]: 387.8–815.6) to 403.0 (95% UI: 272.4–586.6), per 100,000 population. The improvement was of a larger magnitude in women (742.2 to 514.3, per 100,000) compared to men (398.5 to 291.9, per 100,000) (Fig. 1). And we also observed the DALYs in boys younger than 10 years old was larger than that in girls globally (See Supplementary Fig. 1, Additional File 1).

East Asia & Pacific region made the largest progress that the age-standardized DALY rates decreased by 63% (95% UI: 60%-66%), from 378.7 to 138.9 per 100,000. However, the age-standardized rates for DALYs in North America region presented a mild increase of 5% (95% UI: -22%-44%), from a very low level in 1990 (59.3 per 100,000). Sub-Saharan Africa made a small reduction of 15% (95% UI: -6%-21%). We also observed unusually high rates for men in Sub-Saharan Africa region through the decades (572.5 to 562.6, per 100,000) (See Supplementary Table 1, Additional File 1). The magnitude of improvement in women was generally larger than that in men in each region (See Supplementary Table 1, Additional File 1).

### ID burden in 2017 in countries of different HDI

There were 38, 39, 51, and 57 countries in the low-, medium-, high-, and very high-HDI groups. Medians (interquartile ranges, IQRs) of age-standardized DALY rates in these four groups were 686.1 (IQR, 459.3–775.8), 433.9 (IQR, 288.8–549.2), 289.6 (IQR, 137.0–366.2), and 90.5 (IQR, 66.2–134.9) per 100,000, respectively. There were statistically significant differences in age-standardized DALY rates across the four HDI groups (*p* < 0.001): the rate was generally lower in higher HDI groups. However, there was no significant difference between low- and medium-HDI groups (*p* = 0.07) (See Supplementary Fig. 2, Additional File 1).

**Fig. 2:**
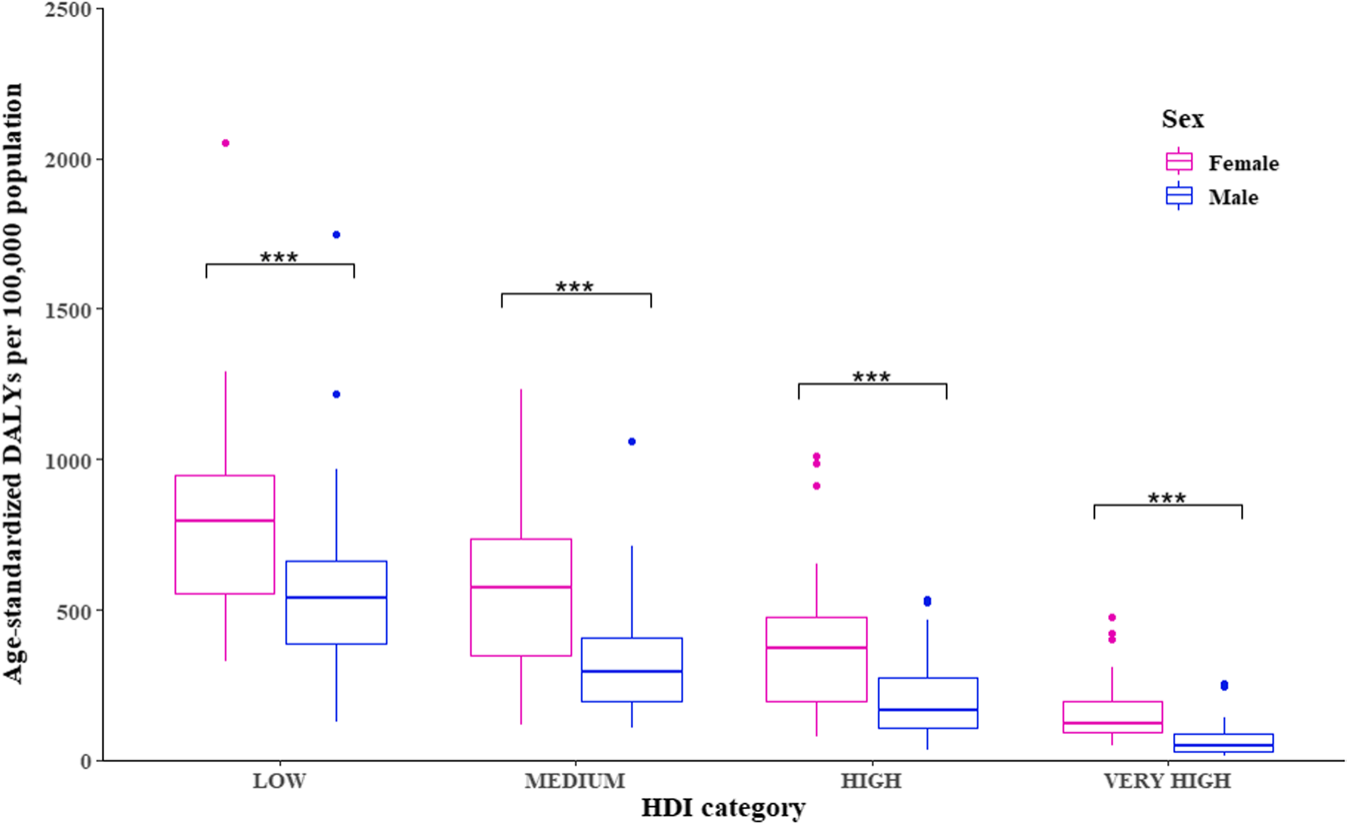
Sex-specific age-standardized DALY rates among countries with different HDI in 2017. Lines inside the boxes indicates the medians; boxes, the 25% and 75% percentiles; and lines outside the boxes, the ± 1·5 times of quantile range; points, outliers. *** *p* < 0.001

Age-standardized DALY rates among women were consistently higher than those among men in each of the four HDI groups: 794.0 (IQR, 552.2–945.25) *vs* 538.0 (IQR, 385.9–663.2) in the low-HDI group, 572.2 (IQR, 346.3–736.7) *vs* 292.9 (IQR, 197.0–406.2) in the medium-HDI group, 372.4 (IQR, 195.9–372.4) *vs* 166.3 (IQR, 109.6–272.4) in the high-HDI group, and 121.5 (IQR, 93.8–195.3) *vs* 31.2 (IQR, 88.8–31.2) in the very high-HDI group, per 100,000 (all *p* < 0.001) (Fig. 2). Linear regression analysis also showed that the gap of ID burden between women and men was negatively associated with HDI (*β* =  − 364.11, *p* < 0.001).

When combining HDI and GDP changes together, we observed that even among countries with similar HDI and had little changes in GDP from 1990 to 2017, there were large differences in the change of their ID burdens (See Supplementary Fig. 3, Additional File 1).

### ID burden in countries of different HDI trajectory clusters

**Fig. 3:**
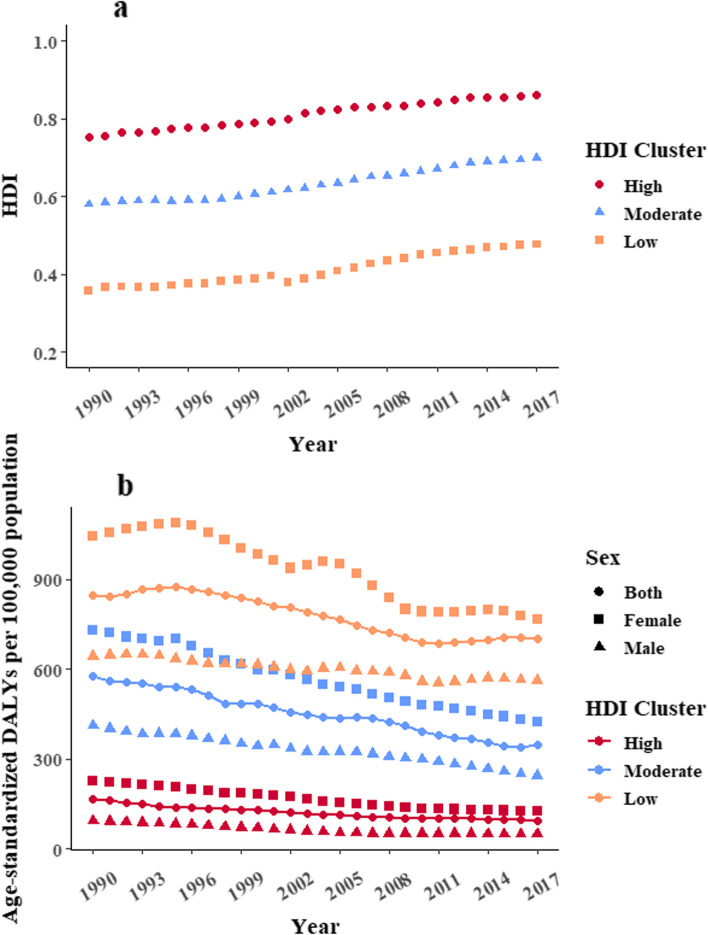
Clustered HDI trends for 152 countries from 1990 to 2017 (**a**). We grouped 23, 77, and 52 countries into each of the low-, moderate- and high-HDI cluster, respectively. Median HDI of each cluster from 1990 to 2017 was plotted. Sex-specific age-standardized DALY rates from 1990 to 2017 with HDI clusters(**b**). 152 countries were included with 23, 77, and 52 countries into each of the low-, moderate- and high-HDI cluster, respectively. Median age-standardized DALY rates from 1990 to 2017 of each cluster were plotted

With HDI data from 1990 to 2017, we were able to cluster 152 countries into three clusters according to changes in HDI across the years. Sine the changes in HDI were relatively stable over the years in all three clusters, we grouped 23, 77, and 52 countries into each of the low-, moderate- and high-HDI cluster, respectively. From 1990 to 2017, the median HDI for low-HDI cluster increased from 0.356 in 1990 to 0.476 in 2017, whereas the increments were from 0.579 to 0.699 and from 0.753 to 0.862 in moderate-HDI and high-HDI clusters, respectively (Fig. 3a).

We observed rather stable ID burden in the high-HDI cluster. However, there was a seemingly decrease in age-standardized DALY rate in the low- and moderate-HDI cluster. Compare to men, decreases in the ID burden among women were more obvious in each cluster, especially in the low- and moderate-HDI clusters. The ID burden in men, on the other hand, did not change substantially over the years, and were consistently high in the low-HDI cluster (Fig. 3b).

### Sex-specific Gini coefficient and concentration index of global ID burden from 1990 to 2017

**Fig. 4:**
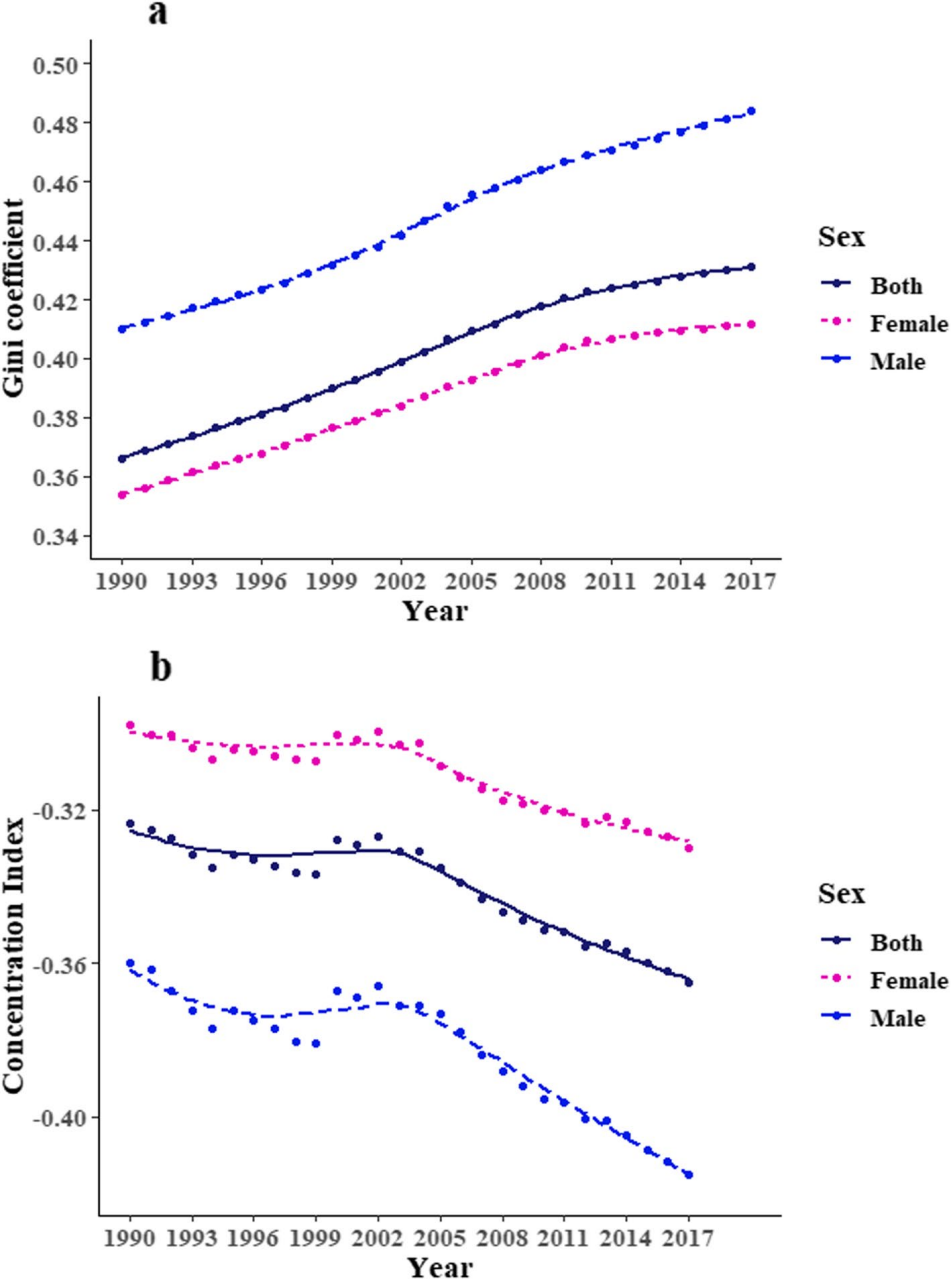
Trends in the Gini coefficients (**a**) and the Concentration indexes (**b**) of ID burden across countries from 1990 to 2017 in both sexes

The Gini coefficient and concentration index were used to measure health inequality. From 1990 to 2017, we observed an increasing trend in the ID inequality globally (*p* < 0.001): the Gini coefficients of ID burden rose from 0.366 to 0.431 (Fig. 4a). If stratified by sex, the Gini coefficient of ID burden rose from 0.354 to 0.411 in women and from 0.410 to 0.484 in men. Concentration index analyses indicated that socioeconomic-associated inequality in ID burden has also increased from 1990 to 2017(− 0.324 to − 0.365), with changes from -0.298 to -0.330 in women, and from -0.360 to -0.415 in men. Both Gini coefficients and concentration indexes suggested that the ID inequality was more severe in men, and worsened faster than that in women. The decrease of concentration index slowed down from 2000 to 2004 and then continued to drop significantly (Fig. 4b).

## Discussion

Although global ID burden has decreased consistently over the decades, we still identified ID inequality in each geographic region and over all, moreover, the inequality has been expanding since 1990. We also observed heavier ID burden in countries with lower HDI, higher ID burden in women than that in men, and smaller between-sex gap of ID burden in countries with higher HDI. Last, the ID inequality seems more severe among men than that in women across the years. To our knowledge, this is among the first studies that examined the overlooked ID inequality issue globally, in different geographic regions, and in each sex.

Although major global efforts have been mounted to address the ID burden in women, sex-related disparity still exists. Despite the narrowed gap of age-standardized DALY rates between women and men, the ID burden has been consistently high in women than that in men. Efforts are still needed to close the gap. Previous studies have found that SES contributes to the lowered ID burden in women, for example, Bentley et al*.*[26] found that high SES is a protective factor for anemia in Indian women, and Bharati et al*.*[27] reported that the lower hemoglobin levels in Indian women than men was partly attributed to sex discrimination. This coincides with our observation where the ID burden for women lowered with higher HDI. Therefore, improving SES in women, e.g., by providing universal education and equal salary, will help reduce ID burden.

We observed rather persistent ID burden among men in countries of low- and medium-HDI groups. Interestingly, we also observed higher ID burden among boys than girls younger than 10 years old. This finding is consistent with a previous multi-country survey conducted in Tanzania, Mozambique, Ghana, Malawi, and Indonesia, where boys who were 12–14 years old were more likely to be anemic than girls of the same age [28]. Gupta et al*.* also observed high ID prevalence in adolescent boys [29].

Previous studies have called for close collaboration between government and schools for both adolescent girls and boys with ID. For example, India started weekly iron-folic acid (IFA) supplementation in selected schools of urban Puducherry in 2012, where the prevalence of anemia was found to be 62.7% [30]. Further, we noticed significantly fewer ID alleviating programs aiming at boys than girls according to the Global database on the Implementation of Nutrition Action (GINA) that monitors nutrition policies and programmes in 202 countries, and the literatures included in a meta-analysis which focused on interventions to improve adolescent nutrition [31]. We can easily identify programmes such as the Girls’ Iron-Folate Tablet Supplementation (GIFTS) program in Ghana, which is aiming at adolescent girls, but it was difficult to identify similarly highlighted programmes for adolescent boys. To close the gap of ID in adolescent boys, we strongly recommend countries that are suffering from high ID burden pay attention to adolescent boys in making their ID alleviation plans.

East Asia & Pacific and South Asia regions have made big strides in controlling ID, while Sub-Saharan Africa countries still need to work out a strategy to lower their ID burden. China is the among the countries in East Asia region that made the most improvement; the age-standardized DALY rate decreased 75% (95% UI: 72%– 78%) from 1990 to 2017. The rapid advancement in the SES over these years may partly explain this improvement; the HDI of China rose from 0.501 to 0.752 during these years. Additionally, governmental actions such as intervention projects may also contributes to the improvement. Wei et al*.* [32] estimated that a 23. 2% decrease in ID prevalence may be attributed to Iron Fortified Soy Sauce Project in China from 2004 to 2013. Other actions, such as Micronutrient Package Project for 6 ~ 24 months infants and Micronutrients Fortified Flour Project may also contribute to alleviation of the ID burden (http://www.chinanutri.cn/FFO/) [33]. Although many iron and folic supplementation programs have been implemented or planned from 1990 to 2027 in South Asia and Sub-Saharan Africa, the ID burden in these regions are very different. (https://extranet.who.int/nutrition/gina/en/map) [34]. In Viet Nam, following a pilot project which distributed weekly iron-folic acid in 2006, together with the de-worming for all women of reproductive age, the prevalence of iron deficiency anemia fell from 38 to 4% at 54 months. Uganda has also implemented public health packages to control ID, but the prevalence of pregnancy anemia remained high and even increased from 41.2% in 2001 to 64.4% in 2006 [35]; a potential reason is the low prevalence of iron/folic acid supplementation use among pregnant women, suggesting the enforcement of such policies may play an important role.

As in countries with high HDI, the link between SES and ID seemed much weaker. Al Zenki et al*. *[36] found that ID prevalence in Kuwait varied by age and gender, but not SES. The majority of very high-HDI level countries seemed to have low ID burdens. On the other hand, even with little changes in GDP, reduction in ID burden can be achieved. For example, Nepal and Bhutan obtained much better progress than most other countries that had similar changes in GDP from 1990 to 2017.

Individual-level SES is closely related to one’s health. According to Phelan et al., individuals and population groups with higher SES are more capable to take advantage of new knowledge, for example, advanced medical treatment [37]. It is quite possible that SES-health gradient, i.e., the disparities in health across SES groups, shift in favor of high SES individuals following the development of new knowledge with empirical evidences [37], which could also happen to ID. There is an educational gradient in ID [38], and enhancing ID-preventing knowledge in people may improve the ID burden [39]. A lot of iron supplementary programs have been adopted in areas with high ID prevalence, but it remained unclear why the disparities in the ID burden is increasing. According to Chang and Lauderdale, when we create intervention that is expensive, complicated, time-consuming to carry out, or difficult to imply broadly, we are likely to create health disparities [40]. We may need to question if current ID interventions are easily to be adopted or need additional resources. For example, adding iron to soy bean sauce or regular food may be more effective than recommending supplements to ID patients.

The current study has a few advantages. First, we analyzed ID burden using the longitudinal GBD data that allowed us to examine the trends in ID burden over decades, globally and in different regions. Second, we used HDI to represent the overall SES of the countries, which is a combination of national income, health and education. Third, we innovatively used Gini coefficient and concentration index to quantify the extent of health inequality.

There are also a few limitations. First, our analyses depended on data from GBD 2017 [41], the validity of the statistical assumptions and data sources used by GBD 2017 may affect our findings. Second, although we provided a global perspective of ID burden and examined its distributions by SES, sex, and geographical regions to discuss ID inequality, we did not include other factors for ID that may also be influential. Therefore, our conclusions may not be generalizable to certain populations. Third, since we used the age-standardized DALY rate as a tool to make the ID burden comparable among different countries, and this metric is not affected by age, we were not able to explore the impact of age in great details.

## Conclusions

We found that the global ID burden has been decreasing since 1990 but the ID inequality kept increasing over the years. There are still heavy ID burden in countries with lower HDI. ID burden was higher in women than that in men, but the ID inequality issue might be more severe in men. Improvements in HDI may shrink the between-sex gap of ID burden, and additional attentions are needed for men, especially for boys in countries with low HDI. However, future studies are needed to examine ID burden and its inequality among specific populations.

## Supplementary Information

**Additional file 1: **

## Data Availability

The datasets generated and/or analyzed during the current study are available in the Human Development Report 2017, http://hdr.undp.org/en/data; Global Health Data Exchange website, http://ghdx.healthdata.org/.

## Abbreviations

ID: Iron deficiency
DALYs: Disability-adjusted life-years
HDI: Human Development Index
SES: Socioeconomic status
GBD 2017: Global Burden of Disease Study 2017
SDGs: Sustainable Development Goals
IQRs: Interquartile ranges
