# Supplementary material for: Global burden and inequality of iron deficiency: findings from the Global Burden of Disease datasets 1990–2017
Source: Nutr J. 2022 Mar 18;21:16. doi: 10.1186/s12937-022-00771-3 (PMC8933942; doi:10.1186/s12937-022-00771-3)

Supplementary Table 1: Sex-specific Age-standardized DALYs Change among World Bank Regions: 1990-2017

|  | Both Sexes | | |  | Female |  |  | Male |  |
| --- | --- | --- | --- | --- | --- | --- | --- | --- | --- |
|  | Age-standardized DALYs  per 10,000 population | | Change | Age-standardized DALYs  per 10,000 population | | Change | Age-standardized DALYs  per 10,000 population | | Change |
|  | 1990 | 2017 |  | 1990 | 2017 |  | 1990 | 2017 |  |
| Global | 569.3  (387.8-815.6) | 403  (272.4-586.6) | -0.29  (-0.32--0.26) | 742.2  (504.3-1052.9) | 514.3  (345.7-740.8) | -0.31  (-0.34--0.27) | 398.5  (266.7-580) | 291.9  (191.3-428.4) | -0.27  (-0.33--0.21) |
| East Asia & Pacific | 378.7  (255.8-551.7) | 138.9  (91.8-206.5) | -0.63  (-0.66--0.6) | 488.9  (332.9-705.8) | 192.6  (127.3-280.4) | -0.61  (-0.64--0.57) | 271.2  (178.6-397.3) | 86.4  (54.6-135) | -0.68  (-0.73--0.63) |
| Europe & Central Asia | 243.5  (158.9-360.9) | 152  (97.9-228.7) | -0.38  (-0.43--0.32) | 301.4  (197-442.9) | 197.6  (129.5-294.6) | -0.34  (-0.4--0.28) | 183.7  (117.8-273.8) | 105.8  (63.5-166.2) | -0.42  (-0.51--0.33) |
| Latin America & Caribbean | 441.5  (290-648.6) | 243.2  (157.6-360) | -0.45  (-0.52--0.37) | 557  (370.3-824.3) | 308.9  (198.4-461.2) | -0.45  (-0.54--0.34) | 318.5  (195.2-492.8) | 172.5  (106.3-273.2) | -0.46  (-0.57--0.33) |
| Middle East & North Africa | 579.5  (387.3-841.1) | 336.1  (220.3-489.3) | -0.42  (-0.47--0.36) | 757.9  (509.6-1091) | 420.7  (280.9-617.4) | -0.44  (-0.49--0.4) | 412.9  (269.7-614.2) | 260.8  (164.2-380.5) | -0.37  (-0.47--0.24) |
| North America | 59.3  (35.8-96.5) | 62.1  (36.5-97.6) | 0.05  (-0.22-0.44) | 73.3  (45.9-118.5) | 76.8  (46.1-120.7) | 0.05  (-0.2-0.36) | 45.5  (24.4-80.3) | 47.3  (22.9-87.7) | 0.04  (-0.43-0.92) |
| South Asia | 1227.9  (832.9-1727.5) | 778.6  (521.6-1113) | -0.37  (-0.41--0.32) | 1743.6  (1189.9-2436.9) | 1093.7  (731.6-1567.4) | -0.37  (-0.41--0.33) | 750.4  (505.7-1064.9) | 471  (309.2-693.7) | -0.37  (-0.44--0.3) |
| Sub-Saharan Africa | 736.2  (494.9-1043) | 628.6  (419.5-913.2) | -0.15  (-0.21--0.06) | 893.5  (604.6-1261.8) | 687  (458.6-995.7) | -0.23  (-0.28--0.18) | 572.5  (385.3-815) | 562.6  (367.9-833.3) | -0.02  (-0.16-0.18) |

*Note.* Numbers were presented as Mean (CI 95%); CI: confidence interval; Change: age-standardized DALYs rates [(2017−1990)/1990]). Data were from GBD Results Tool : <http://ghdx.healthdata.org/gbd-results-tool>

Supplementary Fig.1: Sex-specific DALYs among ages. DALYs=disability-adjusted life years.


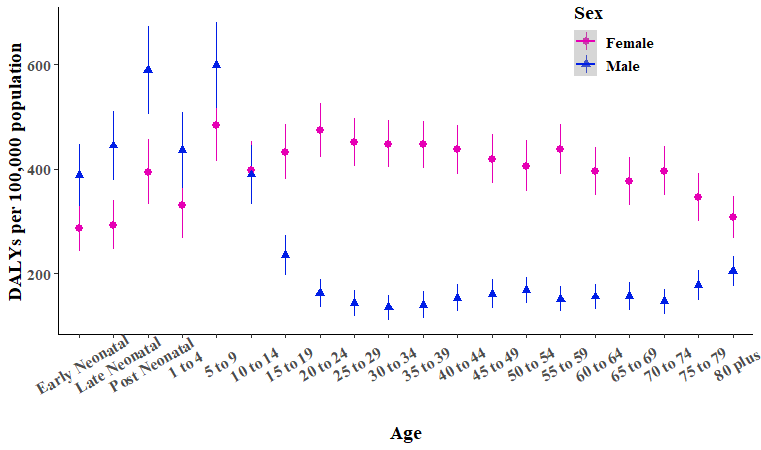


Supplementary Fig. 2: Age-standardized DALY rates among countries with different levels of socioeconomic development in 2017.

HDI=human development index. * *p* < 0.05, *** *p* < 0.001.


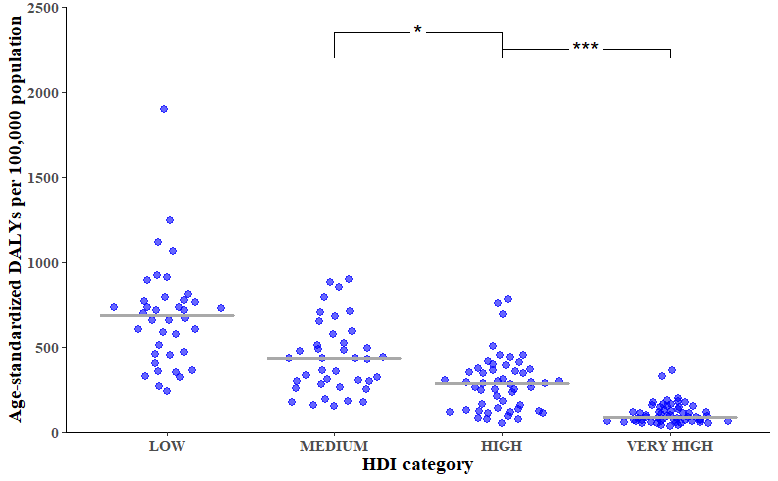


Supplementary Fig. 3: Association between age-standardized DALY rates reduction and GDP growth: 1990-2017. 146 countries are included. HDI levels are grouped with HDI data in 2017; GDP=gross domestic product; Data are in current U.S. dollars.


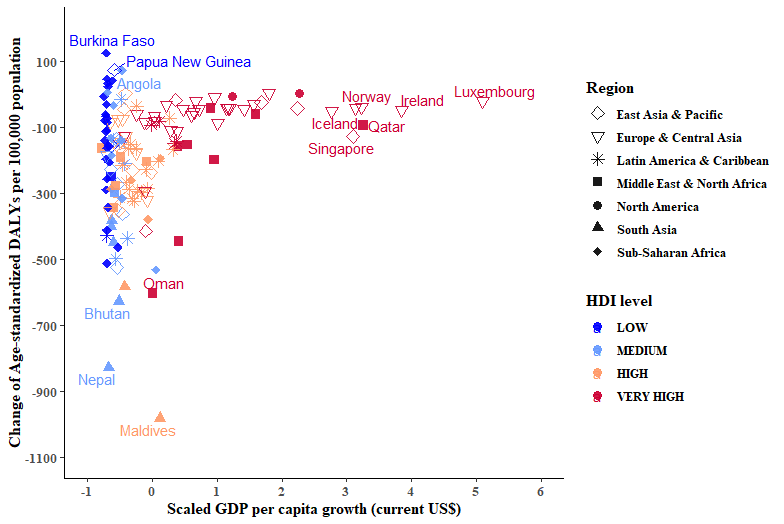

Supplement: Supplementary file 1 — Additional file 1: [file 12937_2022_771_MOESM1_ESM.docx]
